# Structure-guided hydrophobic modulation at the 3-position of *Senecio nutans*–derived chalcones Drives divergent vascular mechanisms

**DOI:** 10.3389/fphar.2026.1772443

**Published:** 2026-05-15

**Authors:** Javier Palacios, Chia Ling Yu González, Diego Aravena, Javier Romero-Parra, Maximiliano Martínez-Cifuentes, Claudio Parra

**Affiliations:** 1 Laboratorio de Bioquímica Aplicada, Química y Farmacía, Facultad de Ciencias de la Salud, Universidad Arturo Prat, Iquique, Chile; 2 Departamento de Química Orgánica y Fisicoquímica, Facultad de Ciencias Químicas y Farmacéuticas, Universidad de Chile, Santiago, Chile; 3 Departamento de Química Orgánica, Facultad de Ciencias Químicas, Universidad de Concepción, Concepción, Chile

**Keywords:** angiotensin-converting enzyme, chalcone, hypertension, Senecio nutans Sch.-Bip., vascular smooth muscle

## Abstract

**Background:**

Hypertension is a major global health challenge driven by sustained alterations in vascular tone and dysregulation of the renin–angiotensin system (RAS). Although natural products provide valuable scaffolds for vascular drug discovery, systematic structure–function studies linking defined hydrophobic modifications to vascular mechanisms remain limited. This study aimed to evaluate how hydrophobic substitution at the three-position of chalcones derived from *Senecio nutans* modulates vascular reactivity and RAS-related pathways.

**Methods:**

A focused series of chalcones bearing either an allyl or a prenyl substituent at the three-position was synthesized and evaluated. Vasorelaxant effects were assessed in isolated aortic rings from spontaneously hypertensive rats under receptor-mediated (phenylephrine), depolarization-induced (KCl), and angiotensin I–induced contractile conditions. Molecular docking studies were performed to explore interactions with the angiotensin-converting enzyme (ACE) catalytic site, and *in vitro* ACE inhibition assays were conducted to support functional findings.

**Results:**

All chalcones induced concentration-dependent relaxation in precontracted aortic rings; however, their pharmacological profiles depended on the nature of the 3-substituent. The prenylated derivative CHAL 13 showed the most consistent activity across complementary vascular models, retaining inhibitory effects under depolarizing conditions and significantly attenuating angiotensin I–induced contraction. In contrast, the allylated derivative CHAL A showed a more limited mechanistic profile: although retaining activity in phenylephrine-precontracted rings, it exhibited weaker inhibition under depolarizing conditions and markedly lower ACE inhibitory potency *in vitro*. Docking studies supported productive accommodation of chalcones within the ACE catalytic pocket, and CHAL 13 showed moderate but significant ACE inhibition *in vitro* (IC_50_ = 20.25 µM), whereas CHAL A was markedly less active.

**Conclusion:**

Hydrophobic substitution at the three-position is a relevant determinant of the vascular behavior of S. nutans–derived chalcones. Prenylation favors a broader vasoactive profile associated with interference in Ca^2+^-dependent contraction and partial modulation of the ACE/Ang II axis. These findings identify CHAL 13 as a promising lead-like scaffold for the development of multifunctional vascular-active agents and support further optimization of prenylated chalcones for antihypertensive drug discovery.

## Graphical Abstract

**Figure:**
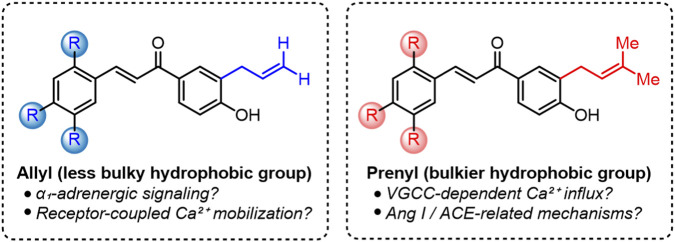

## 1 Introduction

Cardiovascular diseases remain the leading cause of mortality worldwide, accounting for approximately one-third of all global deaths (Di Cesare et al., 2024). Among these disorders, hypertension represents one of the most prevalent and clinically significant risk factors, arising from a multifactorial interplay of genetic, environmental, and lifestyle-related determinants, including age, family history, obesity, physical inactivity, smoking, dietary patterns, and chronic comorbidities (Takase et al., 2025). Current estimates indicate that hypertension affects approximately 1.28 billion adults globally, predominantly individuals between 30 and 79 years of age (Kong et al., 2025), underscoring the urgent need for effective and durable therapeutic strategies.

At the physiological level, blood pressure homeostasis is governed by tightly regulated neurohormonal systems, among which the renin–angiotensin system (RAS) plays a central role in the control of vascular tone, fluid balance, and electrolyte homeostasis (Brewster et al., 2003). Sustained or excessive activation of the RAS contributes to the development and progression of hypertension through persistent vasoconstriction, vascular remodeling, and endothelial dysfunction (Satou et al., 2018). Angiotensin-converting enzyme (ACE) occupies a pivotal position within this cascade by catalyzing the conversion of angiotensin I into the potent vasoconstrictor angiotensin II while simultaneously degrading the vasodilatory peptide bradykinin (Paul et al., 2006). Consequently, pharmacological ACE inhibition, achieved with agents such as captopril, enalapril, fosinopril, and ramipril, remains a cornerstone of antihypertensive therapy (Paul et al., 2006). However, despite their proven clinical efficacy, currently available ACE inhibitors are associated with adverse effects, pharmacokinetic limitations, and tolerability issues that can compromise long-term adherence and therapeutic outcomes (Paul et al., 2006; Manoharan, 2023). These challenges continue to motivate the search for novel molecular entities capable of modulating vascular function through complementary or alternative mechanisms (Manoharan, 2023).

Within this context, natural products continue to represent a rich and chemically diverse source of bioactive scaffolds for drug discovery. Over the past 2 decades, approximately half of newly approved drugs have originated from natural sources or have been inspired by natural product–derived structures (Süntar, 2020). Importantly, increasing emphasis has been placed on the rational transformation of natural metabolites into optimized lead-like compounds through targeted structural modification and structure–activity relationship (SAR) studies. Among South American medicinal plants, *S. nutans* Sch. Beep., an endemic species of the arid Andean region of northern Chile, has attracted attention due to its reported cardiovascular activity (Parra et al., 2018). Previous work from our group demonstrated that hydroalcoholic extracts of *Senecio nutans* significantly reduce arterial blood pressure in both normotensive and spontaneously hypertensive rat models (Cifuentes et al., 2016). These antihypertensive effects were accompanied by cardioprotective actions and a marked reduction of vascular tone in isolated rat aorta, suggesting a direct vasomodulatory mechanism (Cifuentes et al., 2016; Palacios et al., 2023; Paredes et al., 2016).

Chemical investigations of *S. nutans* have identified prenylated acetophenone and benzophenone derivatives as major secondary metabolites (Palacios et al., 2023). Notably, these compounds exhibit significant vasodilatory activity in isolated vascular preparations, supporting their relevance as bioactive scaffolds in vascular pharmacology and their suitability for rational chemical optimization aimed at elucidating SAR trends (Paredes et al., 2016). In line with this approach, we recently reported the synthesis of oxime analogues derived from a natural acetophenone isolated from *S. nutans*, which displayed enhanced ACE inhibitory activity relative to the parent compound (Palacios et al., 2024). These findings highlight the potential of structurally guided modifications of *S. nutans*–derived metabolites to generate compounds with improved vascular and enzymatic profiles.

Chalcones represent a privileged scaffold within natural and semi-synthetic product chemistry, characterized by structural simplicity, synthetic accessibility, and a broad spectrum of biological activities, including anti-inflammatory, anticancer, antimicrobial, antioxidant, and cardiovascular-related effects (Shalaby et al., 2023; Zhai et al., 2022). Several naturally occurring chalcones have been reported to exert vasorelaxant actions through distinct mechanisms. For example, isoliquiritigenin induces endothelium-independent vasodilation via activation of calcium-activated potassium (BK_Ca_) channels (Ye et al., 2019), while dihydrospinochalcone-A and isocordoin isolated from *Lonchocarpus xuul* elicit concentration-dependent relaxation in rat aortic rings (Avila-Villarreal et al., 2013). Despite these advances, systematic comparative evaluations of structurally related chalcones in hypertensive vascular models, particularly studies integrating defined hydrophobic substitutions with mechanistic vascular endpoints, remain scarce.

**FIGURE 1:**
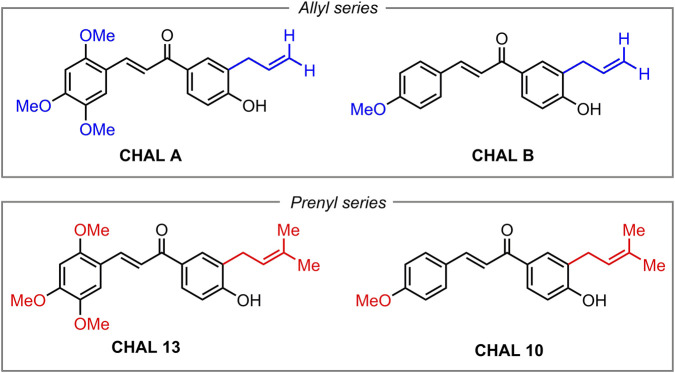
Chemical structures of the chalcones evaluated in this study. CHAL A and CHAL B belong to the allylated series, whereas CHAL 13 and CHAL 10 correspond to their prenylated analogues.

Based on this background, the present study was designed to explore how targeted hydrophobic modulation of a natural product–derived scaffold influences vascular pharmacology in a hypertensive setting. Specifically, a focused series of chalcones derived from acetophenone metabolites of *S. nutans* bearing a prenyl substituent at the three-position was synthesized and compared with structurally related chalcones derived from a commercially available allyl-substituted acetophenone (Figure 1). Using isolated aortic rings from spontaneously hypertensive rats, the compounds were evaluated under receptor-mediated, depolarization-induced, and angiotensin I–dependent contractile conditions. By integrating vascular reactivity assays with *in vitro* enzyme inhibition and molecular modeling, this work aims to delineate structure–activity relationships linking hydrophobic substitution patterns to functional vascular outcomes and ACE-related mechanisms, thereby providing mechanistic insight relevant to the early-stage development of vascular-active small molecules.

## 2 Materials and methods

### 2.1 Synthesis of chalcones

Chalcones were synthesized using a conventional Claisen–Schmidt condensation (Bustos et al., 2022), following established procedures with minor adaptations. Briefly, the appropriate acetophenone and substituted benzaldehyde (1:1 M ratio) were dissolved in ethanol, and an aqueous NaOH solution was added. The reaction mixture was stirred at room temperature for 48 h. Upon completion, the reaction was neutralized with dilute HCl and extracted with ethyl acetate. Organic layers were dried over anhydrous Na_2_SO_4_, concentrated under reduced pressure, and the crude products were purified by column chromatography. Crude products were purified by flash column chromatography on silica gel using hexane/ethyl acetate gradients. Fractions containing the desired products were identified by TLC under UV light and combined prior to solvent removal. Structural identity and purity of all synthesized chalcones were confirmed by ^1^H and ^13^C NMR spectroscopy and HRMS.

(*E*)-1-(4-Hydroxy-3-(3-methylbut-2-en-1-yl)phenyl)-3-(4-methoxyphenyl)prop-2-en-1-one (CHAL 10). A mixture of prenylacetophenone (100 mg, 0.49 mmol) and 4-methoxybenzaldehyde (74 mg, 0.54 mmol) was treated with 20% (w/v) NaOH in methanol (4.9 mL). Purification by column chromatography (0→5→10→25% EtOAc/hexane) afforded chalcone CHAL 10 (50 mg, 32%) as a yellow solid. ^1^H NMR (400 MHz, COSY): δ 7.90 (d, J = 2.1 Hz, 1H, H-β), 7.85 (dd, J = 8.4, 2.2 Hz, 1H, H-6), 7.80 (d, J = 15.6 Hz, 1H, H-2), 7.60 (d, J = 8.8 Hz, 2H, H-2′, H-6′), 7.45 (d, J = 8.8 Hz, 1H, H-α), 6.95 (dd, 3H, H-3′, H-5′, H-5), 5.62 (s, 1H, OH), 5.38 (tt, J = 7.2, 1.3 Hz, 1H, H-2″), 3.86 (s, 3H,OCH_3_), 3.45 (d, J = 7.2 Hz, 2H, H-1″), 1.79 (s, 6H, CH_3_). ^13^C NMR (101 MHz, HSQC): δ 190.0 (CO), 161.6 (C-4′), 159.2 (C-4), 144.3 (C-β), 131.1 (C-3″), 129.0 (C-2′, C-6′), 128.9 (C-2, C-6), 127.8 (C-3, C-1′), 121.6 (C-2″), 119.7 (C-α), 115.8 (C-5), 114.5 (C-3′, C-50), 55.5 (CH_3_O-C4′), 29.2 (C-1″), 25.9 (C-4″), 18.0 (C-5″). The spectral data are identical to those previously reported [10.3390/molecules27144387].

(*E*)-1-(4-Hydroxy-3-(3-methylbut-2-en-1-yl)phenyl)-3-(2,4,5-trimethoxyphenyl)prop-2-en-1-one (CHAL 13). A mixture of prenylacetophenone (100 mg, 0.49 mmol) and 2,4,5-trimethoxybenzaldehyde (106 mg, 0.54 mmol) was treated with 20% (w/v) NaOH in methanol (4.9 mL). Purification by column chromatography (5→10→25% EtOAc/hexane) afforded chalcone CHAL 13 (116 mg, 62%) as a yellow solid. ^1^H NMR (400 MHz, COSY): δ 8.06 (d, J = 15.7 Hz, 1H, H-β), 7.84 (m, J = 7.1 Hz, 2H, H-6 years H2), 7.46 (d, J = 15.7 Hz, 1H, H-α), 7.13 (s, 1H, H-6′), 6.88 (d, J = 9.0 Hz, 1H, H-5), 6.53 (s, 1H, H-3′), 5.62 (s, 1H, OH), 5.35 (t, J = 7.8 Hz, 1H, H-2″), 3.95 (s, 3H, OCH_3_-C5′), 3.91 (s, 6H,OCH_3_-2′), 3.44 (d, J = 7.1 Hz, 2H, C1″), 1.80 (d, J = 5.4 Hz, 6H, CH3). ^13^C NMR (101 MHz, HSQC): δ 189.7 (CO), 158.5 (C-4), 154.5 (C-4′), 152.3 (C-2′), 143.2 (C-β), 139.3 (C-5′), 131.8 (C-3″), 131.1 (C-6), 128.9 (C-2), 126.9 (C-5), 121.2 (C-2″), 120.3 (C-α), 115.8 (C-1′), 115.5 (C-3), 111.5 (C-6′), 96.9 (C-3′), 56.6 (CH_3_O-C5′), 56.4 (CH_3_O-C4′), 56.1 (CH_3_O-C4′), 29.9 (C-1″), 25.8 (C-4″), 18.0 (C-5″). The spectral data are identical to those previously reported [10.3390/molecules27144387].

(*E*)-1-(3-Allyl-4-hydroxyphenyl)-3-(2,4,5-trimethoxyphenyl)prop-2-en-1-one (CHAL A). To a mixture of allylacetophenone (100 mg, 0.568 mmol) and 2,4,5-trimethoxybenzaldehyde (111.8 mg, 0.568 mmol) in a sodium hydroxide/ethanol solution, 20% w/v (10 mL/mmol). Purification by column chromatography (5→10→25→50% EtOAc/hex) gave chalcone (102.5 mg, 51%) as a yellow solid. 1H NMR (400 MHz, COSY): δ 8.07 (d, J = 15.7 Hz, 1H, H-β), 7.86 (s, 2H, H-6, H-2), 7.46 (d, J = 15.7 Hz, 1H, H-α), 7.13 (s, 1H), 6.90 (d, J = 8.7 Hz, 1H), 6.52 (s, 1H), 6.05 (td, J = 16.5, 6.6 Hz, 1H, H-2″), 5.70 (s, 1H, OH), 5.30–5.10 (m, 2H, H-3″), 3.95 (s, 3H, CH3), 3.90 (s, 6H, CH3), 3.49 (d, J = 6.0 Hz, 2H, H-1″). 13C NMR (101 MHz, HSQC): δ 35.3 (C-1″), 56.2 (CH3), 56.5 (CH3), 56.7 (CH3), 97.0, 111.7 (Ph), 115.8 (C-3″), 117.2 (Ph), 120.4 (C-α), 125.5, 129.4, 131.7, 132.0 (Ph), 136.0 (C-2″), 139.6 (C-5′), 143.4 (C-β), 152.5 (C-2′), 154.7 (C-4′), 158.4 (C-4), 189.8 (CO). EI-MS: m/z 355.1497 [M+] (100).

(*E*)-1-(3-Allyl-4-hydroxyphenyl)-3-(4-methoxyphenyl)prop-2-en-1-one (CHAL B). To a mixture of allylacetophenone (100 mg, 0.568 mmol) and 4-methoxybenzaldehyde (77.3 mg, 0.568 mmol) in a sodium hydroxide/ethanol solution, 20% w/v (10 mL/mmol). Purification by column chromatography (10→25→50% EtOAc/hex) gave chalcone (74.6 mg, 45%) as a yellow solid. 1H NMR (400 MHz, COSY): δ 7.87 (s, 2H, H-6, H-2), 7.78 (d, J = 15.5 Hz, 1H, H-β), 7.60 (d, J = 7.7 Hz, 2H, H-2′, H-6′), 7.42 (d, J = 15.6 Hz, 1H, H-α), 6.93 (t, J = 8.3 Hz, 3H, H-5, H-3′, H-5′), 6.04 (m, 2H, H-2″, OH), 5.25–5.15 (m, 2H, H-3″), 3.86 (s, 3H, CH3), 3.49 (d, J = 6.2 Hz, 2H, H-1″). 13C NMR (100 MHz, HSQC): δ 35.2 (C-1″), 55.6 (CH3), 114.5 (Ph), 115.8 (C-3″), 117.2 (Ph), 119.7 (C-α), 125.8, 127.9, 129.4, 130.3, 131.6, 131.7 (Ph), 135.9 (C-2″), 144.2 (C-β), 158.7 (C-4′), 161.7 (C-4), 189.3 (CO). EI-MS: m/z 295.1288 [M+] (100).

### 2.2 Animals

Male spontaneously hypertensive rats (SHR; 4 months old, 180–200 g). Animals were housed under controlled conditions (25 °C, 12 h light/dark cycle) with free access to standard chow and water. Where indicated, data from age-matched normotensive Wistar–Kyoto (WKY) rats obtained were used for comparative purposes. Both strain of rats were obtained from Charles River Laboratories (USA). All experimental procedures were approved by the Animal Ethics Committee of the University of Antofagasta (Protocol CEIC #275/2020) and were conducted in accordance with national and international guidelines for the care and use of laboratory animals.

### 2.3 Aortic tissue preparation

Rats were euthanized via cervical dislocation, and the thoracic aorta was rapidly excised and carefully cleaned of adherent connective tissue (Palacios et al., 2024). Aortic rings (two to three mm in length) were prepared and maintained in Krebs–Ringer solution containing (mM): 118 NaCl, 24 NaHCO_3_, 1 MgSO_4_, 0.435 NaH_2_PO_4_, 5.56 glucose, 1.8 CaCl_2_, and 4 KCl (pH 7.4). Tissues were continuously gassed with 95% O_2_ and 5% CO_2_ and maintained at 37 °C until experimentation.

### 2.4 Vascular reactivity studies

Isometric tension recordings were performed using a DMT 620M wire myograph (Hinnerup, Denmark), following established protocols (Palacios et al., 2018). Aortic rings were mounted on stainless steel wires, set to a basal tension of 1 g, and equilibrated for 30 min in Krebs–Ringer solution. Tissue viability was confirmed by exposure to 60 mM KCl. Vascular responses to phenylephrine (PE; 10^−6^ M), angiotensin I (Ang I; 10^−6^ M), acetylcholine, and test chalcones (CHAL A, CHAL B, CHAL 10, CHAL 13; final concentration 10^−5^ M) were recorded. Chalcones were dissolved in DMSO immediately before use. The final concentration of DMSO in the organ bath did not exceed 0.1% (v/v), a concentration that had no detectable effect on basal tone or vascular reactivity. Data acquisition and analysis were carried out using LabChart 8 software (Colorado Springs, CO, United States).

### 2.5 *In Vitro* angiotensin-converting enzyme inhibition assay

ACE activity was evaluated using a commercial fluorometric assay kit (Darmstadt, Germany) according to the manufacturer’s instructions (Palacios et al., 2025). The assay is based on ACE-mediated cleavage of a synthetic fluorogenic substrate, with fluorescence monitored using a microplate reader (Infinite 200, Tecan, Männedorf, Switzerland) at 320 nm excitation and 405 nm emission. Test compounds (CHAL A and CHAL 13) were assessed over a concentration range of 1–300 µM. Enzyme activity was expressed as a percentage of vehicle-treated controls, and concentration–response curves were generated to calculate IC_50_ values by nonlinear regression. Captopril was included as a reference ACE inhibitor.

### 2.6 Statistical analysis

Data are presented as mean ± standard error of the mean (SEM). Statistical analyses were performed using one-way ANOVA followed by Tukey’s *post hoc* test, with p < 0.05 considered statistically significant. Curve fitting and potency calculations (EC_50_ or pEC_50_) were conducted using GraphPad Prism 8 (San Diego, CA, United States) and SigmaPlot 12.0 (San Jose, CA, United States).

### 2.7 Docking calculations

Docking simulations were performed for the reference ACE inhibitor lisinopril and the synthesized compounds shown in Figure 1. Ligand minimization was carried out using LigPrep in Maestro (Schrödinger v11.8) (Schrödinger Release, 2022, 2018). The crystallographic structure of human ACE (PDB ID: 1O86) (Natesh et al., 2003) was obtained from the RCSB-PDB and prepared with the Protein Preparation Wizard, removing water molecules and assigning ionization states at physiological pH (7.4). Docking calculations were performed using the Glide Induced Fit Docking protocol with the OPLS3e force field and a 26 Å cubic grid centered on the catalytic site defined by the lisinopril binding position (Sherman et al., 2006). Compounds were evaluated using the Glide XP scoring function and filtered based on docking score and RMSD (<1 Å). Binding modes and intermolecular interactions were analyzed using VMD and PyMOL (DeLano, 2002).

## 3 Results and discussion

### 3.1 Effects of 3-substituted chalcones on phenylephrine-induced vascular contraction

**FIGURE 2:**
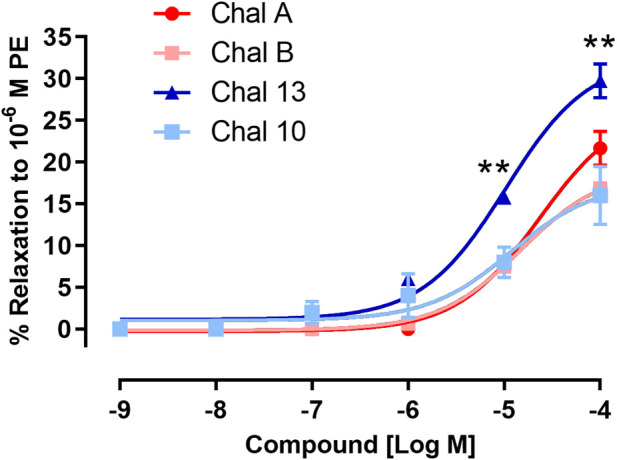
Vasorelaxant effects of chalcones on phenylephrine (PE, 10^−6^ M)-induced contraction in aortic rings from spontaneously hypertensive rats (SHR). Concentration–response curves (% relaxation) for allylated chalcones (CHAL A, CHAL B) and prenylated chalcones (CHAL 10, CHAL 13). Data are expressed as mean ± SEM of four to five independent experiments. **P < 0.01 vs. CHAL13.

To assess receptor-dependent vasorelaxant activity, chalcones were first evaluated in isolated aortic rings from spontaneously hypertensive rats (SHR) precontracted with phenylephrine (PE), an α_1_-adrenergic agonist that induces sustained contraction via receptor-coupled Ca^2+^ mobilization in vascular smooth muscle cells (Henrion et al., 1996). The structures of the evaluated allylated and prenylated chalcones are shown in Figure 1. As shown in Figure 2, all tested chalcones elicited concentration-dependent relaxation responses in PE-precontracted rings, indicating preserved vasorelaxant activity across the series.

Despite this shared qualitative profile, marked differences in efficacy and concentration–response behavior was observed depending on the nature of the hydrophobic substituent at the three-position. Prenylated chalcone (CHAL 13) produced greater maximal relaxation at higher concentrations compared with the allylated derivatives (CHAL A and CHAL B), which displayed reduced efficacy and, in some cases, a decline in relaxation at the upper concentration range. These findings indicate that, under α_1_-adrenergic receptor–dependent conditions, the prenyl substituent confers a functional advantage in terms of maximal vasorelaxant response.

Given the well-documented endothelial dysfunction and heightened smooth muscle reactivity characteristic of SHR aortic tissue, the observed effects are likely mediated predominantly by direct actions on vascular smooth muscle cells rather than by endothelium-dependent mechanisms (Bernatova et al., 2009). From a structure–activity perspective, these results suggest that increased hydrophobic bulk at the three-position, as conferred by the prenyl group, favors maximal vasorelaxant efficacy under receptor-coupled Ca^2+^ signaling conditions. This observation is consistent with a potential role of the prenyl substituent in modulating interactions with lipophilic domains of membrane-associated contractile targets, which are known to be enhanced in hypertensive vascular tissue. In contrast, the allylated derivatives showed reduced maximal relaxation and, in some cases, a decline in response at the upper concentration range, suggesting that the shorter allylic chain may be insufficient to fully engage these targets under α_1_-adrenergic receptor–dependent conditions.

### 3.2 Vasorelaxant effects of chalcones on the contraction

To explore whether the chalcones interfere with receptor-independent Ca^2+^ entry, their effects were evaluated on KCl-induced contraction in aortic rings from spontaneously hypertensive rats (SHR). Elevation of extracellular KCl (60 mM) induces membrane depolarization and subsequent activation of voltage-gated Ca^2+^ channels (VGCCs), predominantly L-type (Ca_V_1.2), and is therefore widely used as a functional model to assess receptor-independent mechanisms of vascular contraction (Ratz et al., 2005).

**FIGURE 3:**
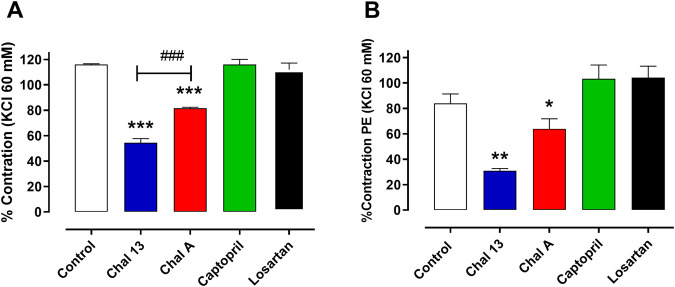
Effects of selected chalcones on KCl- and PE-induced contraction in aortic rings from SHR. Bar graph showing the effect of CHAL A and CHAL 13 on **(A)** KCl (60 mM)-induced contraction and **(B)** PE (10^−6^ M)-induced contraction. Captopril and losartan were included as reference drugs. Data are expressed as percentage of maximal KCl-induced contraction under control conditions and represent mean ± SEM of four to five independent experiments. *P < 0.05, **P < 0.01, ***P < 0.001 vs. control; ###P < 0.001 between indicated groups.

Based on their divergent efficacy profiles under phenylephrine-induced contraction, CHAL A and CHAL 13 were selected as representative allylated and prenylated derivatives, respectively. As shown in Figure 3A, both compounds significantly attenuated KCl-induced contraction compared with control rings. Notably, CHAL 13 produced a significantly greater inhibitory effect than CHAL A, as reflected by a more pronounced reduction in residual contractile response. In contrast, the reference drugs captopril and losartan exerted minimal effects under depolarizing conditions, consistent with their predominantly receptor- and enzyme-dependent mechanisms of action.

These findings are consistent with interference in receptor-independent Ca^2+^ entry, potentially involving voltage-gated Ca^2+^ channels or other membrane-associated processes. Importantly, the greater efficacy of the prenylated derivative CHAL 13 under depolarizing conditions suggests that increased hydrophobic bulk at the three-position may favor interaction with molecular targets associated with Ca^2+^ influx or with the surrounding lipid environment. However, direct interaction with specific Ca^2+^ channel subtypes was not evaluated in the present study. Future studies employing L-type Ca^2+^ channel antagonists such as nifedipine, or direct measurements of intracellular Ca^2+^, will be required to determine whether CHAL 13 directly modulates voltage-gated Ca^2+^ channels. Since α_1_-adrenergic receptors predominate in vascular smooth muscle cells (Docherty, 2019), the direct effect of chalcones on vascular smooth muscle was assessed stimulating alpha-adrenergic receptor with phenylephrine dose-response curves in aortic rings pre-incubated in the presence of a submaximal concentration of chalcones. As shown Figure 3B, CHAL 13 and CHAL A produced a significant reduction in the contractile response to PE, likely by affecting calcium release from sarcoplasmic reticulum and calcium influx from extracellular, as previously described with KCl.

### 3.3 Vasorelaxant effects on angiotensin I–induced contraction

To explore the potential involvement of the renin–angiotensin system (RAS), the effects of chalcones on angiotensin I (Ang I)-induced contraction were evaluated in aortic rings from spontaneously hypertensive rats (SHR). Ang I is enzymatically converted to angiotensin II by angiotensin-converting enzyme (ACE), which subsequently activates AT_1_ receptors, leading to Ca^2+^ mobilization and increased vascular tone (Triebel and Castrop, 2024; Paul et al., 2006).

**FIGURE 4:**
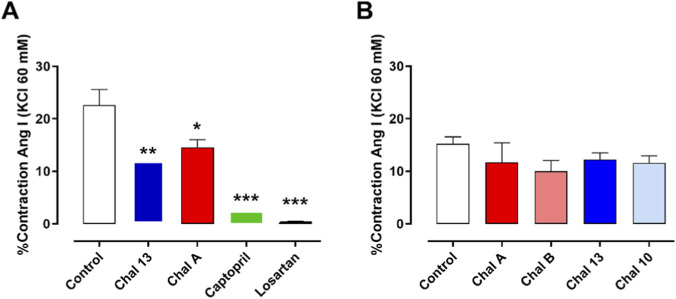
Effects of chalcones on Angiotensin I–induced contraction in aortic rings from SHR. **(A)** Percentage of contraction induced by Ang I (10^−6^ M) under control conditions and in the presence of CHAL A, CHAL 13, captopril, or losartan. **(B)** Effects of additional chalcones (CHAL A, CHAL B, CHAL 13, CHAL 10) on Ang I-induced contraction. Data are expressed as percentage of maximal Ang I-induced contraction and represent mean ± SEM of four to five independent experiments. *P < 0.05, **P < 0.01, ***P < 0.001 vs. control.

As shown in Figure 4, Ang I-induced contraction was significantly attenuated in the presence of CHAL A and CHAL 13 compared with control rings, with the prenylated derivative CHAL 13 producing a more pronounced inhibitory effect. This response profile closely paralleled that observed for the reference compounds captopril and losartan, which almost completely abolished Ang I-induced contraction, thereby confirming the functional involvement of the ACE/Ang II/AT_1_ axis in this experimental model. Because Ang I-induced contraction depends on sequential conversion of Ang I to Ang II by ACE, followed by activation of AT1 receptors and downstream Ca^2+^-dependent signaling, the reduced contractile response observed with CHAL 13 is compatible with interference at one or more levels of the ACE/Ang II/AT1 pathway. The *in vitro* ACE assay supports ACE as one contributing target; however, the present experiments do not exclude additional actions downstream of Ang II formation, including modulation of AT1 receptor signaling or associated Ca^2+^-dependent contractile mechanisms.

Integration of the PE-, KCl-, and Ang I-based assays reveals a differentiated mechanistic profile among the chalcones. While allylated derivatives exhibited higher efficacy under α_1_-adrenergic receptor–dependent conditions, the prenylated compound CHAL 13 retained inhibitory activity under membrane depolarization and Ang I stimulation, suggesting a dual mode of action involving both Ca^2+^ influx modulation and interference with RAS-related pathways. Given the endothelial dysfunction characteristic of SHR, these effects are most likely mediated by direct actions on vascular smooth muscle cells (Paredes et al., 2016).

### 3.4 Molecular docking analysis of chalcones at the angiotensin-converting enzyme

**FIGURE 5:**
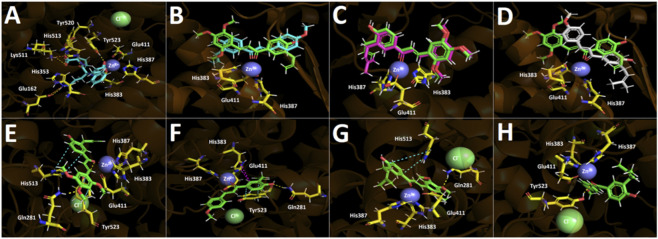
Predicted intermolecular interactions in the human Angiotensin-converting enzyme (ACE) catalytic site. Yellow dotted lines indicate hydrogen bond interactions, cyan dotted lines represent π-π interactions, magenta dotted lines represent T-shaped interactions, red dotted lines represent coordination bond interactions and blue dotted lines represent salt bridges. **(A)** ACE inhibitor Lisinopril binding mode; **(B)** Superimposed binding poses of compounds CHAL A (green) and CHALB (cyan) within the ACE catalytic site; **(C)** Superimposed binding poses of compounds CHAL A (green) and CHAL 13 (magenta) within the ACE catalytic site; **(D)** Superimposed binding poses of compounds CHAL A (green) and CHAL 10 (white) within the ACE catalytic site; **(E)** Binding mode of compound CHAL A; **(F)** Binding mode of compound CHAL B; **(G)** Binding mode of compound CHAL 13; **(H)** Binding mode of compound CHAL 10.

To gain molecular insight into the differential inhibition of Ang I–induced contraction, docking studies were performed to evaluate the binding of chalcones within the catalytic site of angiotensin-converting enzyme (ACE). Simulations were conducted for CHAL A, CHAL B, CHAL 10, and CHAL 13, using lisinopril as a reference ACE inhibitor. As shown in Table 1, lisinopril showed the most favorable binding energy (−11.660 kcal/mol), consistent with its extensive network of stabilizing interactions, including six hydrogen bonds with Glu162, Glu384, Lys511, His513, Tyr520, and Tyr523, three ionic interactions involving Lys511, Glu384, and Glu162, a π-cation interaction with His353, and coordination with Zn^2+^ (Figure 5A).

**TABLE 1:** Binding energies (kcal/mol) of chalcones and lisinopril in the ACE catalytic site.

Compound	Binding energy (kcal/mol) Angiotensin-converting enzyme (ACE)
CHAL A	−8.661
CHAL B	−7.050
CHAL 13	−8.296
CHAL 10	−8.031
Lisinopril	−11.660

The chalcone derivatives displayed less favorable but overall comparable binding energies, with CHAL A showing the best profile (−8.661 kcal/mol), followed by CHAL 13 (−8.296 kcal/mol), CHAL 10 (−8.031 kcal/mol), and CHAL B (−7.050 kcal/mol). In general, the chalcones adopted similar binding poses within the ACE catalytic pocket (Figures 5B–H), although CHAL B showed an inverted orientation relative to the other derivatives, which likely accounts for its poorer energetic profile. A particularly close similarity was observed between CHAL A and CHAL 13, whose docking poses were nearly superimposable, differing only by a slight angular deviation. Likewise, CHAL A and CHAL 10 shared an overall similar disposition within the enzymatic pocket, although CHAL 10 exhibited a more pronounced deviation, which led to the loss of favorable contacts and, consequently, a less favorable binding energy.

Among the chalcones, CHAL A showed the best docking performance. Its binding mode is characterized by a hydrogen bond with Gln281 involving the oxygen atom of the methoxy group located para to the α,β-unsaturated carbonyl system, a π–π interaction with His513 through its phenolic ring, and a coordination interaction with Zn^2+^ (Figure 5E). In addition, CHAL A establishes two further contacts with Tyr523, namely, a hydrogen bond and a π–π interaction, which are absent in the closely related derivative CHAL 13 (Figure 5F). These additional contacts likely contribute to the slightly more favorable binding energy observed for CHAL A (Figure 5B).

CHAL 13 retained the main interactions observed for CHAL A, including the interaction with Gln281, the π–π contact with His513, and the coordination with Zn^2+^ (Figure 5G). Notably, the predicted Zn^2+^ coordination distances for CHAL A and CHAL 13 were 2.1 and 2.2 Å, respectively, values comparable to that calculated for lisinopril (2.2 Å; Supplementary Table S1, Supplementary Material). However, due to its slightly tilted binding orientation relative to CHAL A, CHAL 13 does not establish the two additional interactions with Tyr523 (Figure 5C). This subtle difference in binding geometry may account for its somewhat less favorable energetic profile, despite its overall similar pose and strong predicted affinity. Importantly, this docking behavior is consistent with the biological results, since both CHAL A and CHAL 13 showed the most pronounced inhibition in the vascular contraction assays.

Finally, CHAL 10 exhibited an intermediate binding profile. Its docking pose revealed a hydrogen bond between the chalcone carbonyl group and Tyr523, together with a coordination interaction involving the same carbonyl group and Zn^2+^ (Figure 5H). While these contacts support productive binding, the overall orientation of CHAL 10 was less favorable than that of CHAL A (Figure 5D) and CHAL 13, which may explain its lower affinity compared with these two derivatives.

**FIGURE 6:**
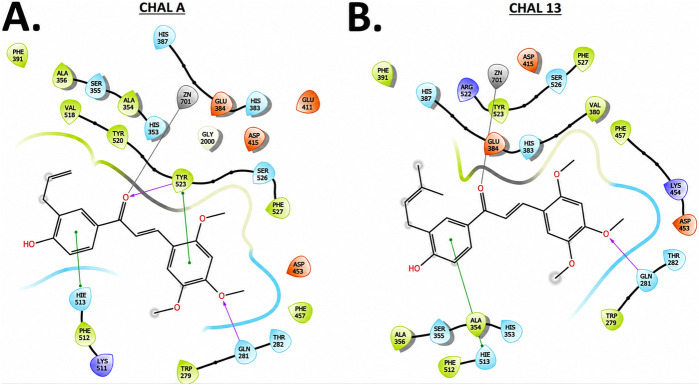
Two-dimensional interaction diagrams for CHAL A **(A)** and CHAL 13 **(B)** in the ACE catalytic site. Key residues are indicated as follows: green (hydrophobic), orange (negatively charged), sky blue (polar), and purple (positively charged).

Overall, the docking analysis suggests that the differences in inhibitory activity among the chalcone derivatives can be rationalized by their distinct binding orientations within the ACE catalytic site and by the nature and number of their interactions with key residues. In particular, CHAL A and CHAL 13, both possessing three methoxy substituents on an aromatic ring (Figures 6A,B), displayed the most favorable docking profiles within the ACE catalytic site and showed the greatest inhibitory activity in the vascular contraction assays. It is noteworthy, however, that CHAL A exhibited a marginally better docking score than CHAL 13 (−8.661 vs. −8.296 kcal/mol), yet substantially weaker inhibitory potency in the biochemical ACE assay (IC_50_ = 130.64 vs. 20.25 µM, respectively; Table 2). This apparent discrepancy between computational and experimental data likely reflects the limitations of static docking simulations, which do not account for conformational flexibility, differential solvation effects, or protein-induced fit dynamics upon ligand binding.

**TABLE 2:** *In vitro* ACE inhibitory activity of selected chalcones.

Compound	IC_50_ ACE (µM)
CHAL 13	20.25 ± 0.09^a^
CHAL A	130.64 ± 0.16^b^
Captopril*	0.005 ± 0.001^c^

* Used as positive control; Values having different superscripts differ significantly (p < 0.05).

### 3.5 *In vitro* inhibition of angiotensin-converting enzyme

Based on their divergent functional effects in the Ang I–induced contraction assay, CHAL A and CHAL 13 were selected as representative compounds for biochemical evaluation of angiotensin-converting enzyme (ACE) inhibition. In agreement with both the functional vascular data and the docking predictions, the prenylated chalcone CHAL 13 inhibited ACE activity *in vitro* with an IC_50_ value of 20.25 ± 0.09 µM, whereas the allylated analogue CHAL A exhibited substantially weaker inhibition (Table 2). Captopril, used as a reference inhibitor, displayed an IC_50_ in the low nanomolar range, consistent with previously reported values (Palacios et al., 2025).

Although CHAL 13 is markedly less potent than captopril, the clear difference in ACE inhibitory activity between the prenylated and allylated chalcones supports a structure-dependent modulation of the ACE/Ang II axis. Importantly, this moderate biochemical inhibition is consistent with the partial, rather than complete, suppression of Ang I–induced contraction observed in vascular assays, suggesting that ACE inhibition likely contributes to, but does not fully explain, the vascular effects of CHAL 13, which may additionally involve downstream RAS signaling and/or direct modulation of Ca^2+^-dependent contractile pathways. Together, these findings suggest that prenylation at the three-position may favor interaction with ACE and thereby contribute to the preferential attenuation of Ang I-mediated vascular responses, positioning this scaffold as a preliminary lead for further optimization rather than as a direct ACE inhibitor. Additional studies evaluating the effect of CHAL 13 on Ang II-induced contraction or AT1 receptor-dependent responses will be necessary to define the relative contribution of ACE inhibition versus downstream RAS signaling.

## 4 Conclusion

The present study demonstrates that hydrophobic modulation at the three-position strongly influences the vascular profile of *S. nutans*–derived chalcones in aortic tissue from spontaneously hypertensive rats. Although all compounds preserved vasorelaxant activity, the nature of the hydrophobic substituent determined the breadth and consistency of the response across the different contractile models evaluated.

Among the tested compounds, the prenylated derivative CHAL 13 showed the most robust and mechanistically informative profile. In addition to relaxing precontracted vessels, CHAL 13 remained active under KCl-induced depolarization and significantly attenuated angiotensin I–induced contraction, supporting an effect that extends beyond a single receptor-dependent pathway. This behavior is compatible with interference in Ca^2+^-dependent contractile processes and suggests that prenylation may favor interactions with membrane-associated targets involved in vascular smooth muscle contraction.

Complementary docking and biochemical data indicate that ACE is one relevant contributor to this activity. Although CHAL 13 was markedly less potent than captopril, its moderate *in vitro* ACE inhibition, together with its effect on angiotensin I–induced contraction, supports partial modulation of the ACE/Ang II axis as part of its vascular mechanism. At the same time, the incomplete inhibition observed in functional assays indicates that ACE inhibition alone does not fully explain its activity and that additional downstream or parallel mechanisms are likely involved.

Taken together, these findings establish a well-defined structure–activity relationship within this focused chalcone series and identify prenylation as a favorable modification for expanding vascular activity toward a multifunctional profile. Accordingly, prenylated CHAL 13 emerge as promising lead structure for further optimization aimed at the development of new antihypertensive agents with combined effects on vascular smooth muscle reactivity and RAS-related signaling.

## Data Availability

The original contributions presented in the study are included in the article/Supplementary Material, further inquiries can be directed to the corresponding authors.
