# Supplementary material for: Structure-guided hydrophobic modulation at the 3-position of Senecio nutans–derived chalcones Drives divergent vascular mechanisms
Source: Front Pharmacol. 2026 May 15;17:1772443. doi: 10.3389/fphar.2026.1772443 (PMC13219365; doi:10.3389/fphar.2026.1772443)

Structure-Guided Hydrophobic Modulation at the 3-Position of *Senecio nutans*–Derived Chalcones Drives Divergent Vascular Mechanisms

Javier Palacios[1], Chia Ling Yu González[1], Diego Aravena[1], Javier Romero-Parra[2], Maximiliano Martínez-Cifuentes[3,*], Claudio Parra[3,*]

[1]Laboratorio de Bioquímica Aplicada, Facultad de Ciencias de la Salud, Universidad Arturo Prat, Iquique 1110939, Chile.

[2]Departamento de Química Orgánica y Fisicoquímica, Facultad de Ciencias Químicas y Farmacéuticas, Universidad de Chile, Santiago 6640022, Chile.

[3]Departamento de Química Orgánica, Facultad de Ciencias Químicas, Universidad de Concepción, Edmundo Larenas 129, Concepción 4070371, Chile.

* Correspondence: maxmartinez@udec.cl (M.M.-C.) and cparra@udec.cl (C.P.)

Experimental

General

All reactions were carried out under an argon atmosphere using dry, freshly distilled solvents under anhydrous conditions. Analytical thin-layer chromatography (TLC) was performed on SiO_2 plates (Merck silica gel 60 F_{254}), and spots were visualized using a 1% aqueous $KMnO_4$ solution. Flash column chromatography was carried out on SiO_2 (SDS silica gel 60 ACC, 35–75 µm, 230–240 mesh ASTM). Organic extracts were dried over anhydrous $MgSO_4$ unless otherwise stated. Solvent evaporation was performed using a rotary evaporator. NMR spectra were recorded in $CDCl_3$ on a Varian VNMRS 400 spectrometer. Chemical shifts for ^1H and ^{13}C NMR spectra are reported in parts per million (ppm) downfield (δ) relative to tetramethylsilane (TMS).

1-(4-Hydroxy-3-(3-methylbut-2-en-1-yl)phenyl)ethan-1-one

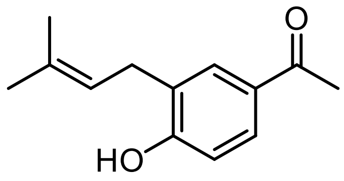

From 180.00 g of dried *Senecio nutans*, 12.97 g of crude extract were obtained by maceration in ethanol. The extract was fractionated by column chromatography using SiO_2 as the stationary phase and a gradient of EtOAc/hexane (5→10→25%), affording the target acetophenone with minor impurities. Recrystallization yielded 0.54 g of a white solid. This procedure was repeated until a total of 6.61 g of the compound was obtained. ^1H NMR (400 MHz, COSY): δ 7.77 (1H, s, H-6'), 7.74 (1H, s, H-2'), 6.86, (1H, d, J =8,1 Hz, H-5), 6.27 (1H, s ancho, OH), 5.31 (1H, t, J =6,9 Hz, H-2'), 3.40 (2H, d, J =6,9 Hz, H-1'), 2.55 (3H, s, H_3CCO), 1.78 (6H, s, CH_3) ^{13}C NMR (100 MHz, HSQC): δ 17.9 (C-4'), 25.8 (C-5'), 26.3 (CH_3CO), 29.6 (C-1'), 115.5 (C-5), 121,0 (C-2'), 127.0 (C-3), 128.9 (C-2), 130.2 (C-1), 130.8 (C-6), 135.4 (C-3'), 159.1 (C-4), 197,5 (CO). Spectral data are identical to those previously reported[1].

General Methodology A: Synthesis of Chalcones

A mixture of acetophenone (1.0 equiv.) and the corresponding substituted benzaldehyde (1.1 equiv.) were dissolved in 20% (w/v) NaOH in methanol (10 mL/mmol). The reaction mixture was stirred at room temperature for 48 h. After completion, the reaction was neutralized with

5% HCl and extracted with EtOAc (3 × 50 mL). The combined organic layers were dried over anhydrous Na_2SO_4, filtered, and concentrated under reduced pressure. The crude product was purified by column chromatography using EtOAc/hexane mixtures of increasing polarity.

Non-Invasive Blood Pressure Measurement

Systolic blood pressure was measured by tail-cuff plethysmography using a BIOPAC system (USA). Briefly, an occlusion cuff was placed around the tail and inflated to 140–200 mmHg. Systolic pressure was determined from the reappearance of the pulse signal during cuff deflation. Data were recorded and analyzed using AcqKnowledge software (BIOPAC Systems, USA).

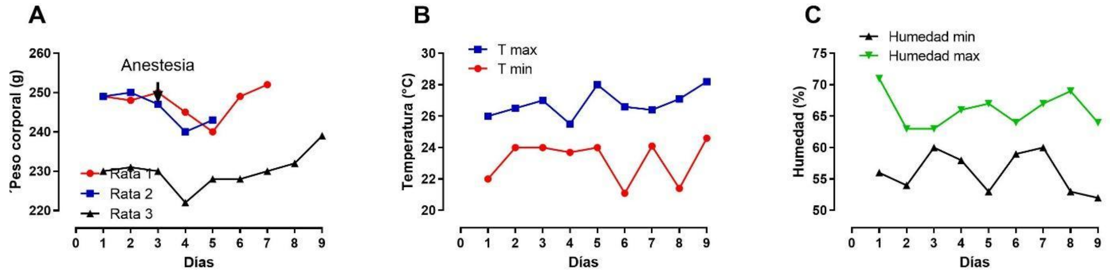

Figure S1. Monitoring of body weight and environmental conditions of SHR rats over time. (A) Body weight. (B) Ambient temperature: maximum (blue) and minimum (red). (C) Ambient humidity: maximum (green) and minimum (black). In panel A, on day 3, the rats were anesthetized to perform an electrocardiogram. Data represents three independent experiments, $n = 3$. (Data were collected up to the time of animal sacrifice.)

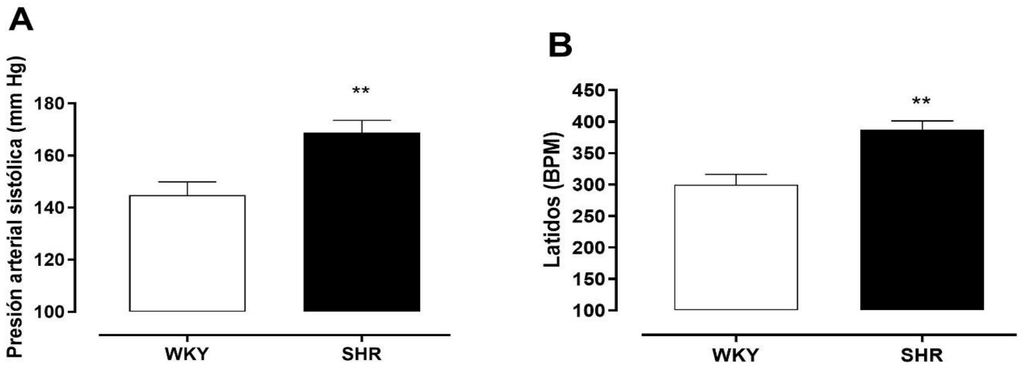

Figure S2. Increase in systolic blood pressure and heart rate in hypertensive and control animals. (A) Systolic blood pressure (mm Hg) and (B) Heart rate (BPM) in conscious spontaneously hypertensive rats (SHR) and normotensive Wistar-Kyoto rats (WKY). Data represents three independent experiments, $n = 3$. **p < 0.01 vs. normotensive (WKY) group.

Electrocardiography (ECG)

Electrocardiographic recordings were obtained as previously described[3]. Rats were anesthetized with ketamine (42 mg/kg) and xylazine (5 mg/kg), and electrodes were positioned in a standard lead II configuration. Heart rate, systolic blood pressure, P–R interval, corrected Q–T interval (QTc), and sympathovagal balance (LF/HF ratio) were recorded and analyzed using AcqKnowledge software.

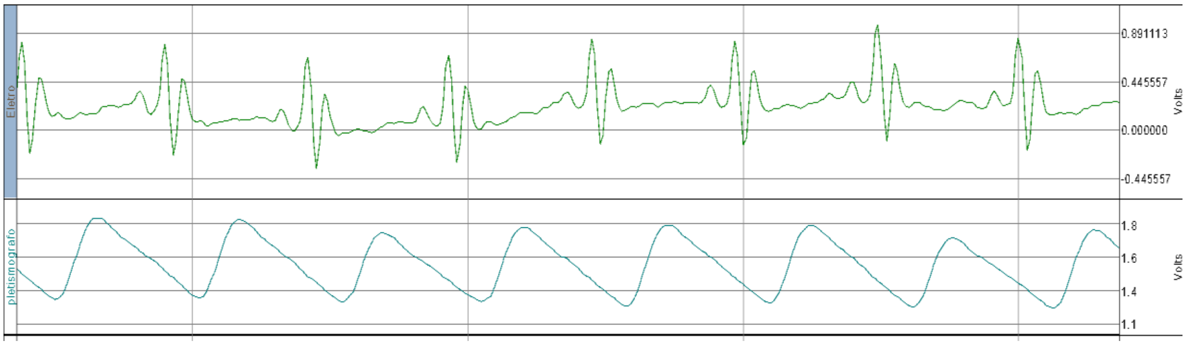

Figure S3. Representative electrocardiogram (ECG) of an anesthetized spontaneously hypertensive rat (SHR).

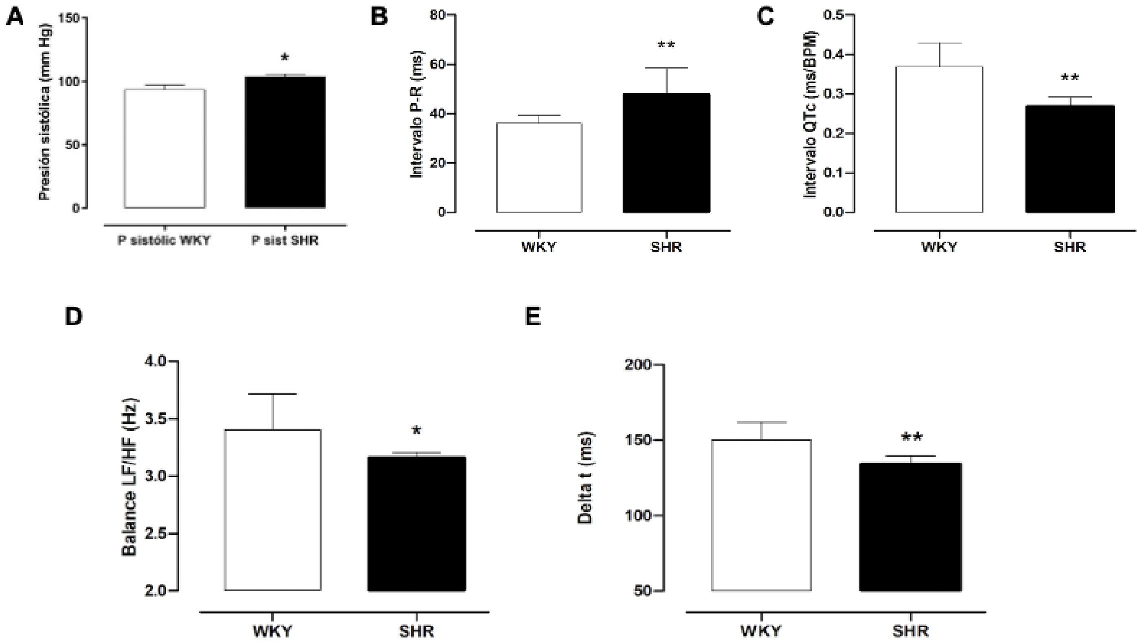

Figure 4S. Cardiovascular characteristics of anesthetized spontaneously hypertensive rats (SHR) and normotensive Wistar-Kyoto rats (WKY). Cardiovascular parameters assessed: (A) Systolic blood pressure, (B) PR interval, (C) Heart rate-corrected QT interval (QTc), (D) Sympathovagal balance expressed as LF/HF ratio, and (E) Latency of the pulse wave from the heart to the tail artery (Delta t). Data represents three independent experiments, $n = 3$. $p < 0.05$; **p** < 0.01 vs. control (WKY).

Table S1. Selected residue-level interaction analysis of CHAL A, CHAL 13, and lisinopril within the ACE catalytic site, including interacting residues, ligand atoms, interaction type, and interatomic distances.

Ligand	Residue ACE	Ligand Atom	Interaction Type	Distance (Å)
CHAL 13	His383	O carbonyl	proximity catalytic region	3.2
CHAL 13	Tyr523	aromatic ring	π–π stacking	3.7
CHAL 13	Gln281	phenolic OH	hydrogen bond	2.1
CHAL 13	His383/Glu411	O carbonyl	coordination/proximity Zn^{2+}	2.2
CHAL A	Tyr523	allyl group	weak hydrophobic contact	6.2
CHAL A	His383/Glu411	O carbonyl	coordination/proximity Zn^{2+}	2.1
Lisinopril	His383/Glu411	carboxylate	coordination/proximity Zn^{2+}	2.2

References

1. Parra, C., Soto, E., León, G., et al. (2018). Nutritional composition, antioxidant activity and isolation of scopoletin from *Senecio nutans*: support of ancestral and new uses. *Natural Product Research*, **32**(6), 719–722.
2. Bustos, L., Echiburú-Chau, C., Castro-Alvarez, A., et al. (2022). Cytotoxic effects on breast cancer cell lines of chalcones derived from a natural precursor and their molecular docking analysis. *Molecules*, **27**(14): 4387.
3. Palacios, J., Fonseca, J., Ayavire, F., et al. (2018). Ascorbate attenuates oxidative stress and increased blood pressure induced by 2-(4-hydroxyphenyl)amino-1,4-naphthoquinone in rats. *Oxidative Medicine and Cellular Longevity*, **2018**: 8989676.

^{1}H NMR (400 MHz, CDCl$_3$)

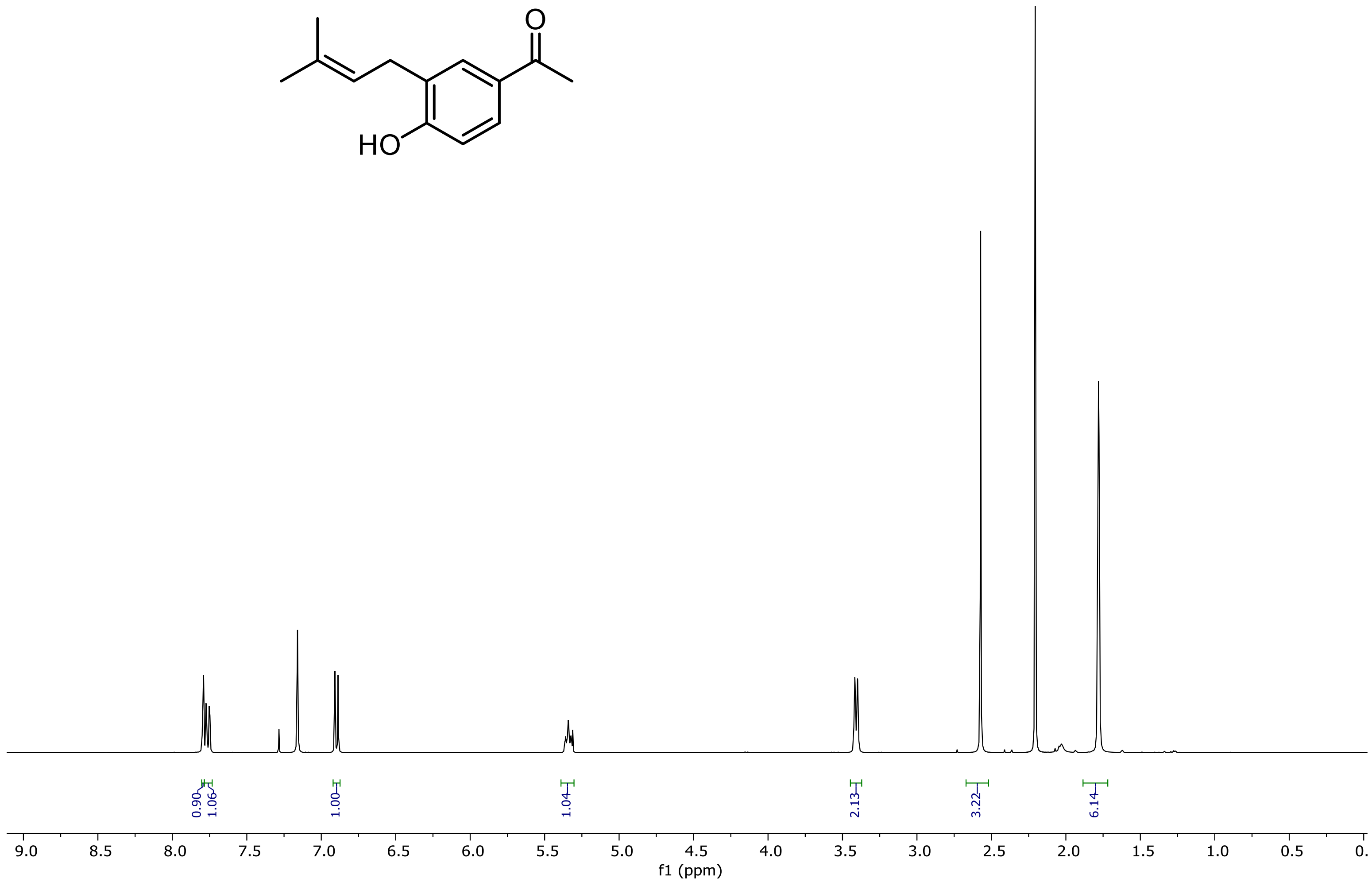

^{13}C NMR (100 MHz, $CDCl_3$)

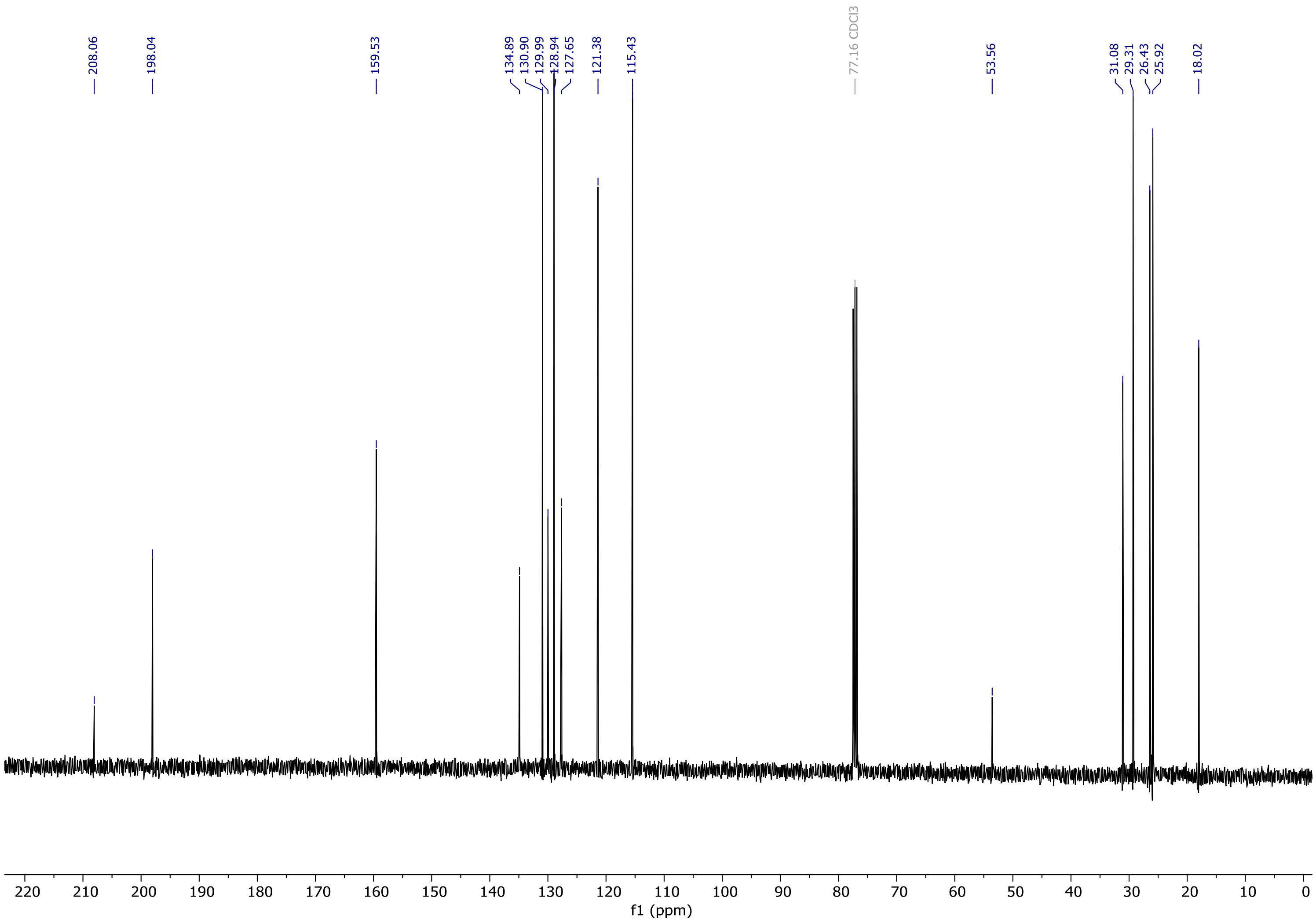

^{1}H NMR (400 MHz, $CDCl_3$)

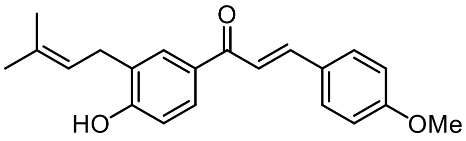

CHAL 10

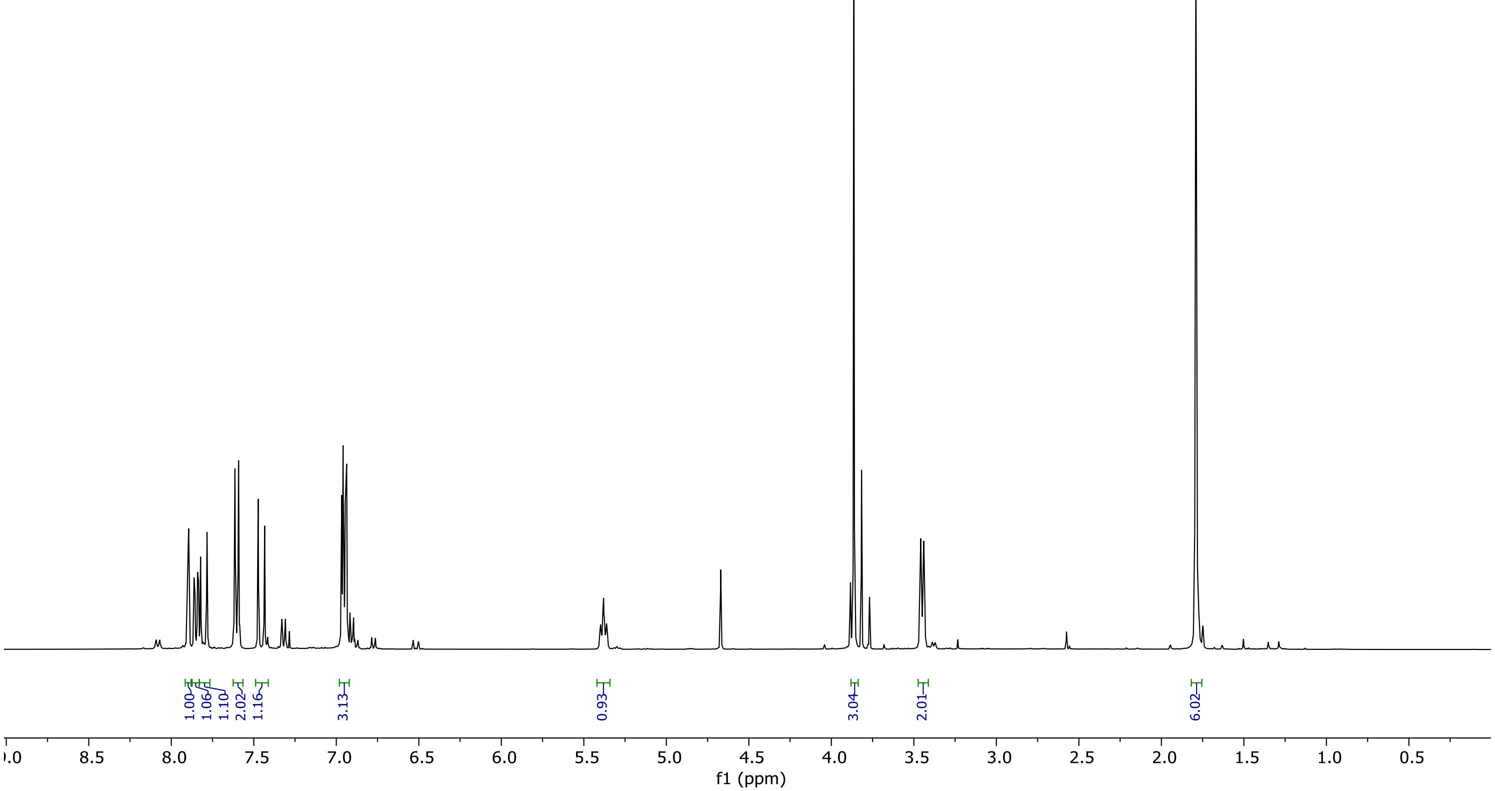

^{13}C NMR (100 MHz, $CDCl_3$)

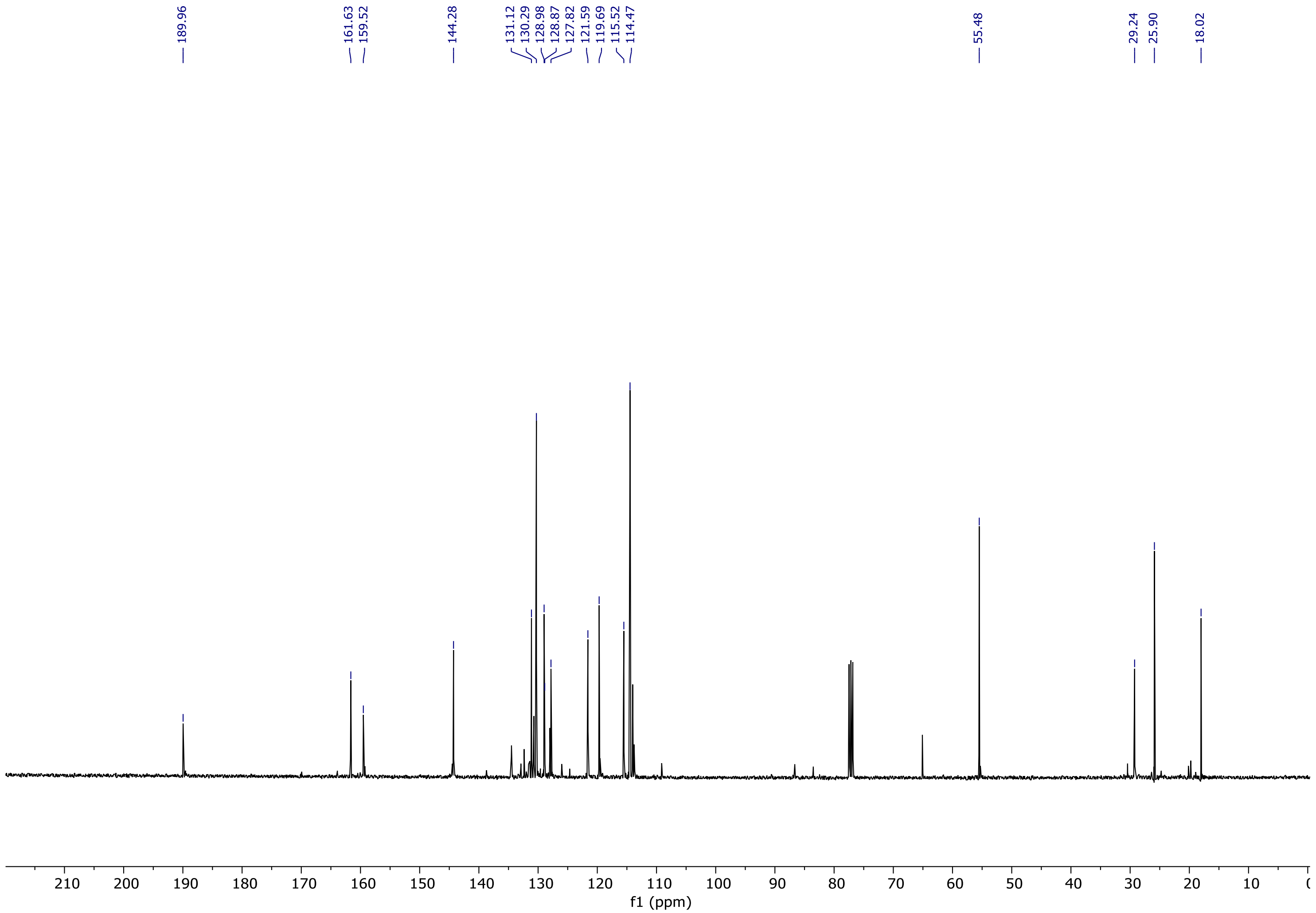
189.96
161.63
159.52
144.28
131.12
130.29
128.98
128.87
127.82
121.59
119.69
115.52
114.47
55.48
29.24
25.90
18.02
210 200 190 180 170 160 150 140 130 120 110 100 90 80 70 60 50 40 30 20 10
f1 (ppm)

^{1}H NMR (400 MHz, CDCl$_3$)

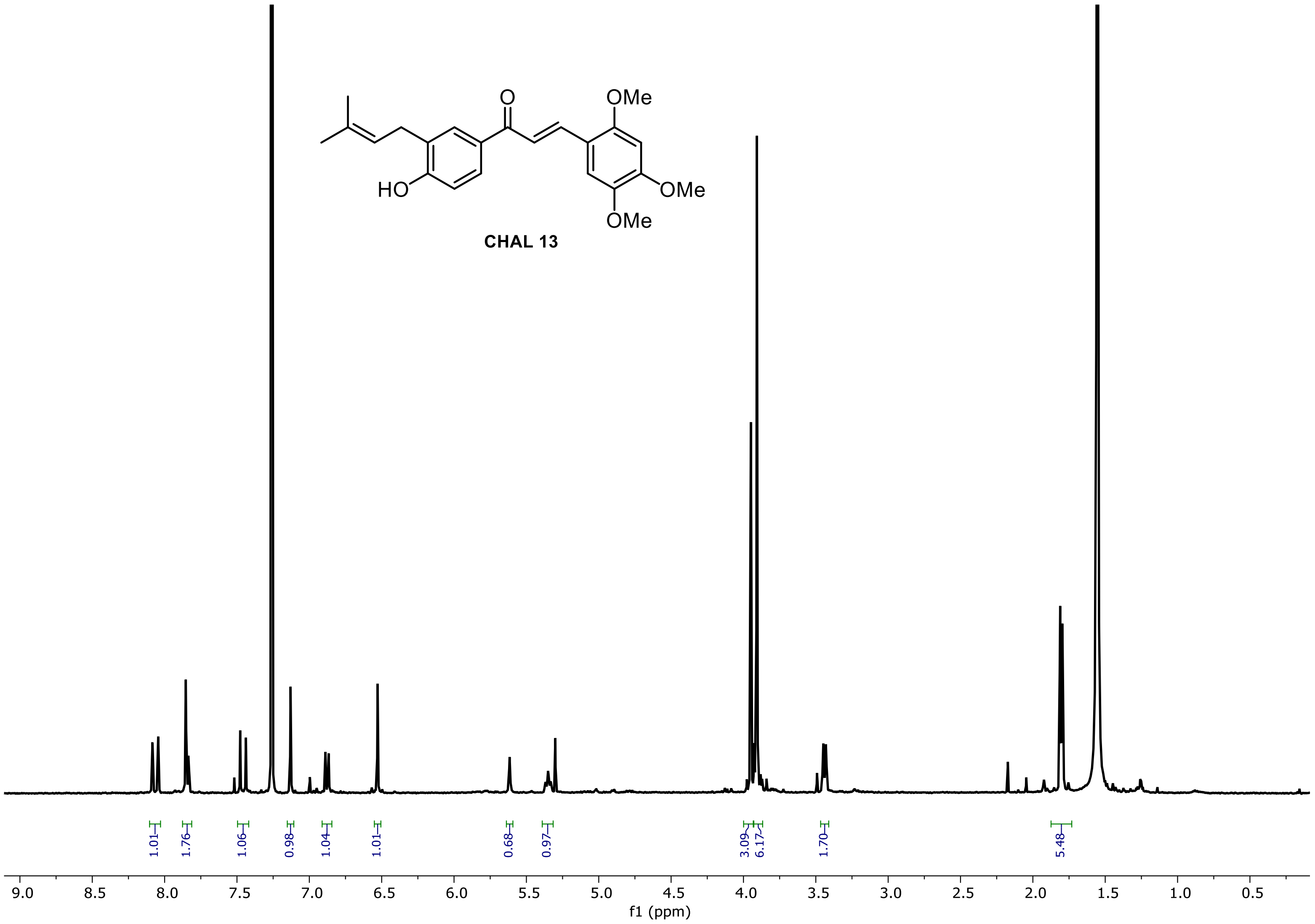

^{13}C NMR (100 MHz, $CDCl_3$)

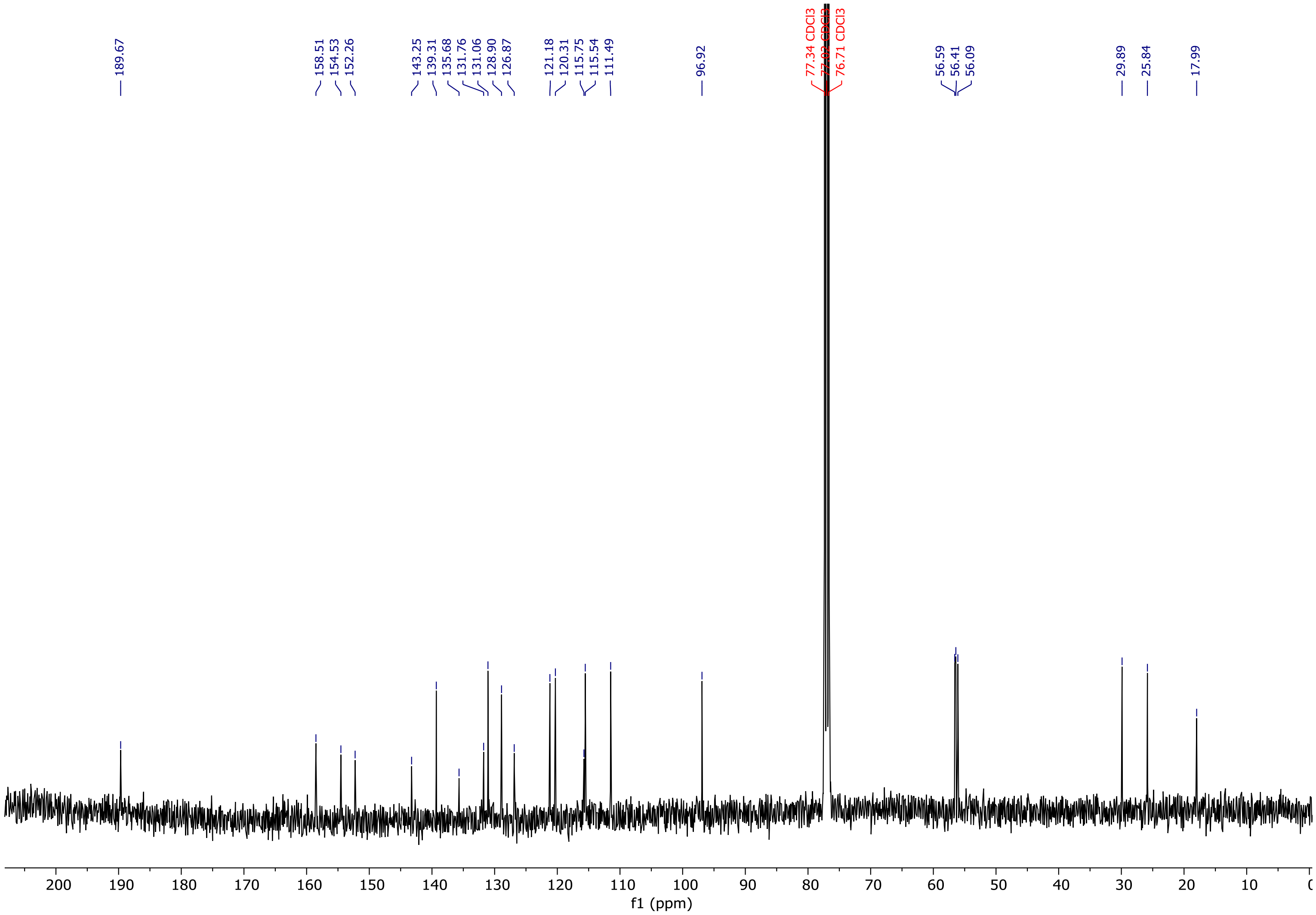

^{1}H NMR (400 MHz, $CDCl_3$)

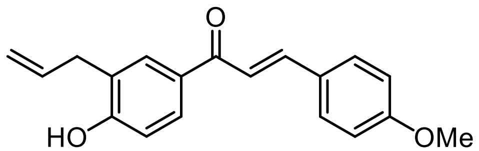

CHAL A

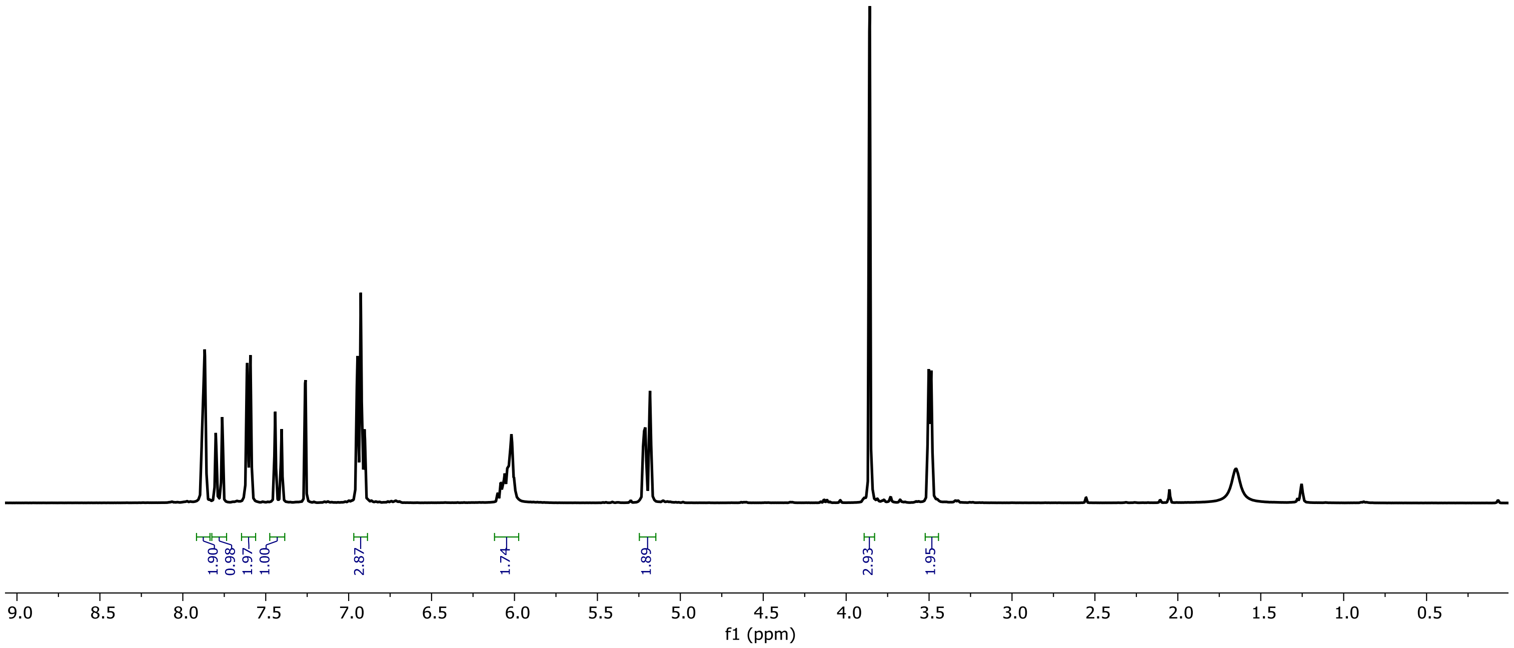

^{13}C NMR (100 MHz, $CDCl_3$)

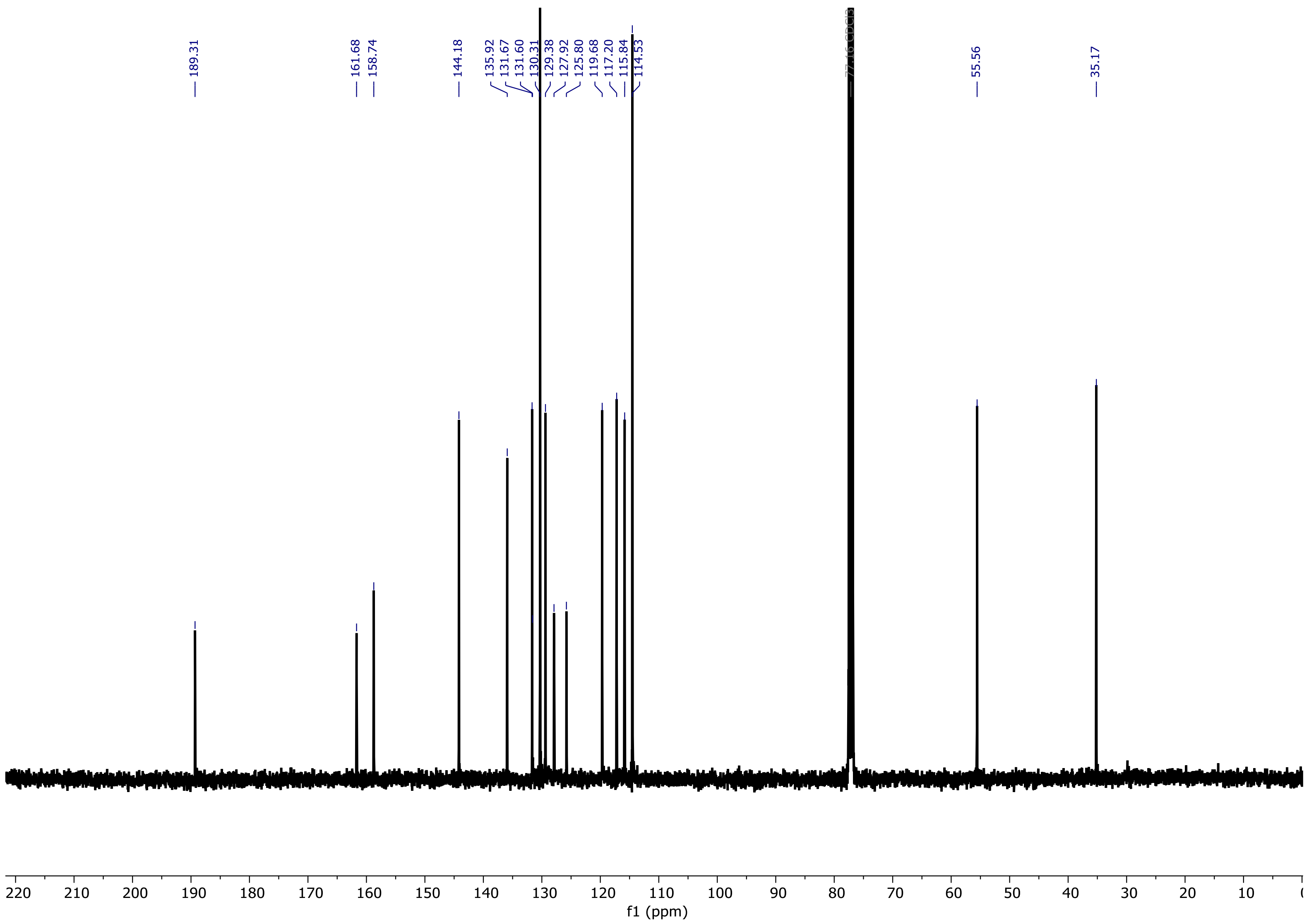

^{1}H NMR (400 MHz, $CDCl_3$)

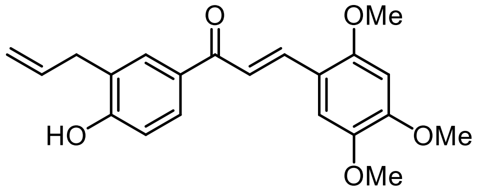

CHAL B

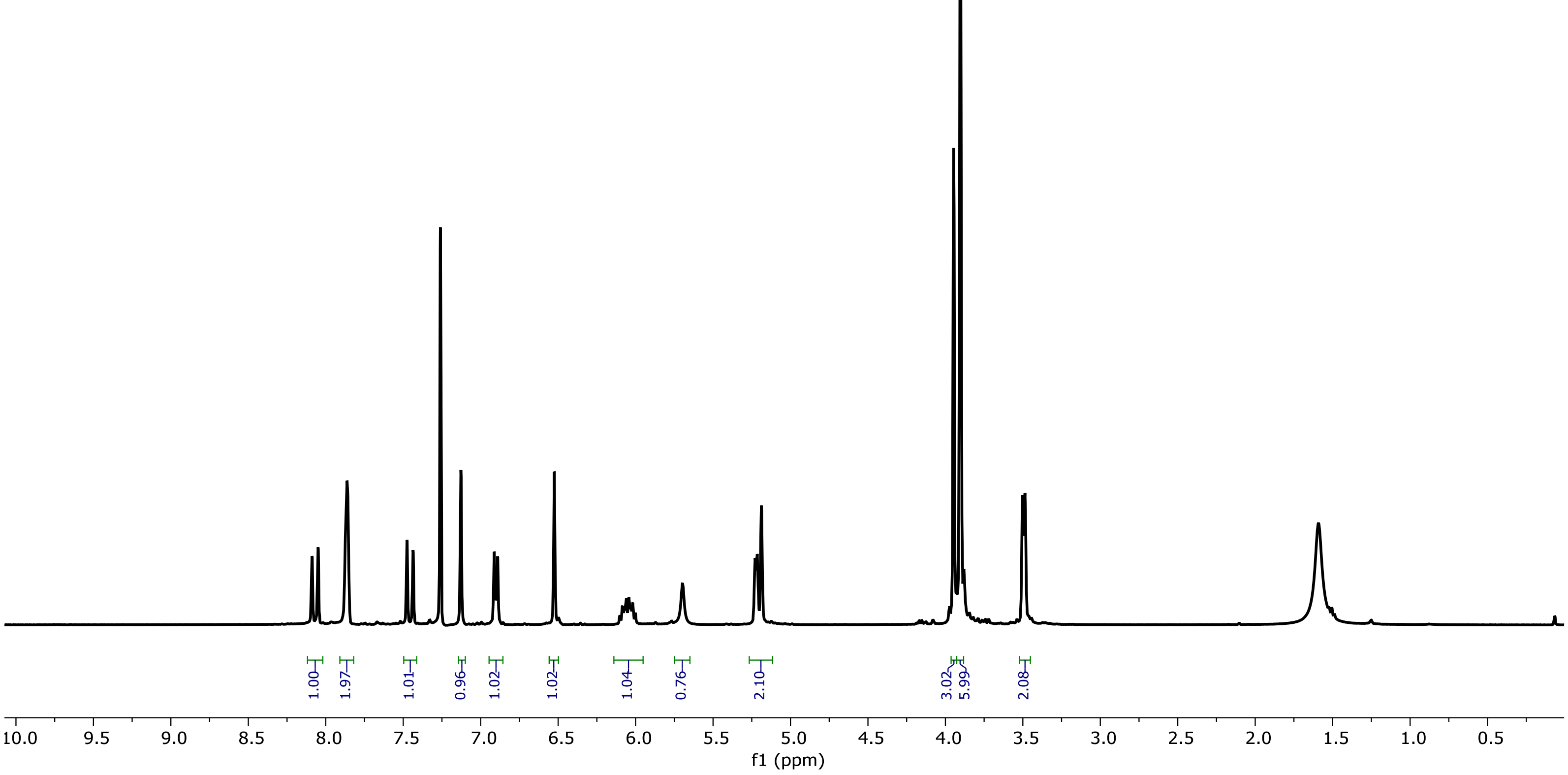

^{13}C NMR (100 MHz, $CDCl_3$)

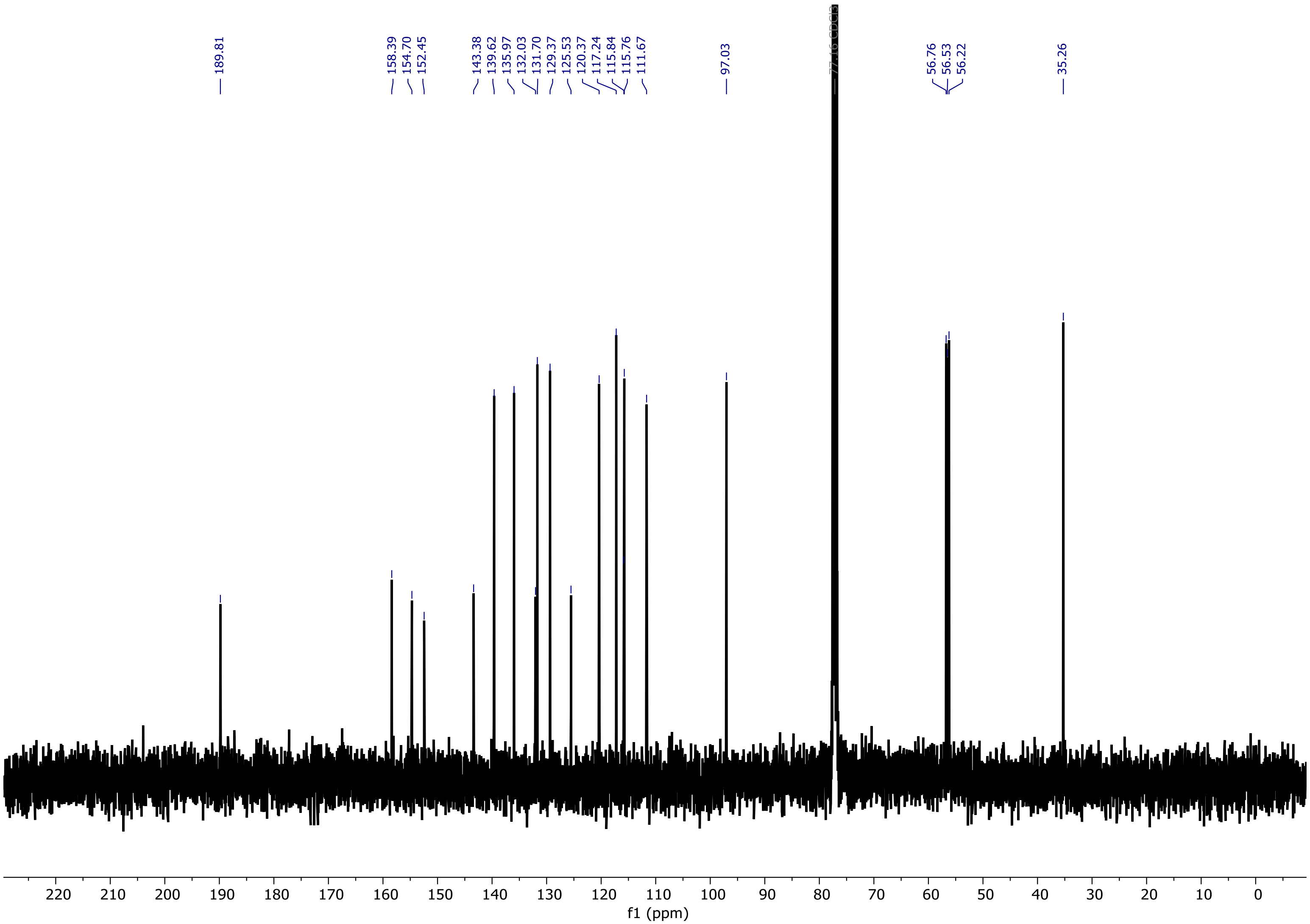

Supplement: Supplementary file 1 [file Supplementaryfile1.pdf]
